# Supplementary material for: Macromammalian faunas, biochronology and palaeoecology of the early Pleistocene Main Quarry hominin-bearing deposits of the Drimolen Palaeocave System, South Africa
Source: PeerJ. 2016 Apr 18;4:e1941. doi: 10.7717/peerj.1941 (PMC4841245; doi:10.7717/peerj.1941)
Supplement: Table S1 [file peerj-04-1941-s001.pdf]

Supplemental Table 1. Catalogue of the indeterminate bovid craniodental and postcranial remains from the Drimolen Main Quarry deposits.

DN Specimen	Element Side	Size Class	Element Description
Craniodental			
28	-	III?	Enamel fragment
31	Right	I/II	Mandibular m3; Likely representing Tribe Antilopini or Tribe Oreotragini
47	-	II	Horn core fragment; Likely representing Tribe Antilopini
48	-	II/III	Horn core tip fragment
92	Left	I/II	Mandibular i1
110	Left	I	Horn core with partial pedicle and orbital rim; Likely Tribe Neotragini or Tribe Oreotragini
124	-	-	Maxillary molar fragment
127	-	-	Enamel fragment
158	Left	I	Maxillary M1 or M2; Likely representing Tribe Oreotragini
176	Right	I	Maxillary P2; Likely Tribe Neotragini or Tribe Oreotragini
192	-	II	Horn core fragment
311	Right	I/II	Mandibular i2
379		I	Enamel fragment
433	Right	I	Mandibular m3; Likely Tribe Oreotragini
434	Right	I	Mandibular m1 or m2; Likely Tribe Antilopini or Tribe Oreotragini
450	Left	II	Mandibular i1
454	-	II/III	Horn core fragment
460	Left	III	Mandibular i2
466	-	I	Horn core fragment; Likely Tribe Neotragini, Tribe Oreotragini or Tribe Peleini
478	-	I	Enamel fragment
497	Left	I	Mandibular i1
567	-	-	Enamel fragment
611	Right	-	Mandibular i3 or canine
626	-	-	Enamel fragment
629	-	-	Enamel fragment
647	-	II	Maxillary molar fragment
649	-	II	Enamel fragment
684	-	-	Enamel fragment

DN Specimen	Element Side	Size Class	Element Description
766	-	II	Horn core fragment
774	-	III	Horn core fragment
782	Right	I	Maxillary premolar; Likely Tribe Oreotragini
791	-	II/III	Horn core fragment
874	-	-	Horn core fragment
900	-	II	Horn core tip fragment
924	-	II	Horn core tip fragment
952	-	I	Mandibular molar fragment; Likely Tribe Antilopini or Tribe Oreotragini
999	Left?	II	Horn core fragment; Possibly Tribe Reduncini
1004	Left	II	Maxillary molar frag; Possibly Tribe Tragelaphini
1005	Left	II	Mandibular i2
1009	-	-	Horn core fragment
1024	-	II/III	Horn core tip fragment; Possibly Tribe Alcelaphini
1036	-	I	Mandibular m1; Likely Tribe Antilopini or Tribe Oreotragini
1070	-	II/III	Horn core fragment
1076	Right	III	Mandibular i2
1085	-	-	Enamel fragment
1086	Left	II	Mandibular diastema from near symphysis to under premolar region
1166	Left	II/III	Two horn core fragments; no articulations
1188	-	-	Enamel fragment
2028	-	II/III	Horn core fragment
2048	-	II/III	Horn core fragment
2054	-	II/III	Horn core tip fragment
2055	-	I/II	Horn core tip fragment
2069	-	II	Horn core fragment; Possibly female *Antidorcus recki*
2077	-	I	Horn core fragment; Likely Tribe Neotragini, Tribe Oreotragini, or Tribe Peleini
2079	-	II	Isolated basisphenoid, unfused suture for basioccipital
2084	-	II	Horn core fragment with pedicle (sinus present); Likely juvenile
2096	-	I/II	Horn core fragment
2107	-	I/II	Horn core fragment
2108	-	II/III	Horn core fragment

DN Specimen	Element Side	Size Class	Element Description
2132a	Left	II	Mandibular i1; 2132a-f associated remains from the same individual
2132b	Left	II	Mandibular i2; 2132a-f associated remains from the same individual
2132c	Left	II	Mandibular i3; 2132a-f associated remains from the same individual
2132d	Left	II	Mandibular canine; 2132a-f associated remains from the same individual
2132e	Right	II	Mandibular i2; 2132a-f associated remains from the same individual
2132f	Right	II	Mandibular i3; 2132a-f associated remains from the same individual
2178	Right	II	Mandibular i3 or canine
2237	-	I/II	Horn core fragment
2238	-	-	Horn core fragment
2257	-	II/III	Horn core tip fragment; Possibly Tribe Alcelaphini or Tribe Reduncini
2269	Right	II	Partial temporal bone with os petrosum and auditory bulla and external auditory meatus
2269	Right	II	Partial inferior orbital rim with temporal process
2269	-	II	Partial neurocranial vault fragment
2299	-	I/II	Horn core fragment; Possibly Tribe Antilipoini, Tribe Reduncini, or Tribe Alcelaphini
2320	Right	II	Mandibular i3 or canine
2332	-	-	Enamel fragment
2391	-	II/III	Horn core fragment
2420	-	-	Possible horn core fragment
2433	Left	II	Mandibular i1
2434	Right	II	Mandibular i3 or canine
2469	Left?	II	Isolated auditory bulla
2473	Right	II/III	Three horn core fragments; no articulations
2499	-	I/II	Horn core fragment
2504	-	II/III	Horn core fragment
2508	-	II/III	Horn core fragment
2542	Right	I	Mandibular m2; Possible Tribe Peleini
2557	-	-	Mandibular enamel fragment; Probably from deciduous p4
2566	-	-	Horn core fragment
2582	-	II/III	Horn core fragment
2582	-	II/III	Horn core fragment with pedicle preserved; Possibly Tribe Reduncini
2835	Right	1	Maxillary deciduous P3; Likely Tribe Antilopini, Tribe Neotragini, or Tribe Oreotragini

DN Specimen	Element Side	Size Class	Element Description
2891	-		Enamel fragment
2906	-	-	Horn core fragment
2920	-	-	Enamel fragment
2921	-	-	Enamel fragment
2926	-	-	Enamel fragment
2932	-	-	Two specimens; Enamel fragment and diaphysis fragment (possibly indet. side tibia)
2942	-	II/II	Three horn core fragments; No articulations
2959	-	II/III	Horn core fragment
2974	Right	I	Mandibular i2
2976	Left	II	Isolated occipital condyle fragment
2990	-	II/III	Horn core tip fragment
2991	-	-	Horn core fragment
3001	-	-	Enamel fragment
3025	-	II/III	Three enamel fragments enamel frags; No articulations
3210	-	-	Two horn core fragments; no articulations
3045	Left	I	Mandibular diastema portion
3055	-	-	Enamel fragment
3061	-	II/III	Enamel fragment
3083	Left	II	Mandibular i3 or canine
3092	-	II/III	Enamel fragment
3097	-	II/III	Enamel fragment
3123	-	-	Enamel fragment
3131	-	II/III	Horn core fragment
3141	-	-	Enamel fragment
3145	-	-	Enamel fragment
3160	-	-	Enamel fragment
3167	Right	I	Maxillary P2; Likely Tribe Neotragini or Tribe Oreotragini
3168	-	-	Enamel fragment
3173	-	-	Enamel fragment
3187	-	II/III	Horn core fragment
3193	-	-	Horn core fragment

DN Specimen	Element Side	Size Class	Element Description
3194	-	I/II	Horn core fragment
3201	-	-	Enamel fragment
3205	-	II/III	Enamel fragment
3206	-	-	Enamel fragment
3043	Right	-	Five specimens; three horn core fragments (two II/III) and two indeterminate taxon fragments
3228	-	-	Enamel fragment
3230	-	-	Enamel fragment
3232	-	-	Horn core fragment
3239	-	-	Enamel fragment
3242	-	-	Two specimens; horn core fragment and possible horn core fragment
3245	-	-	Cranial vault fragment with suture preserved
3262	-	I/II	Enamel fragment
3292	-	-	Horn core fragment
3316	-	-	Horn core fragment
3321	-	-	Enamel fragment
3351	-	I	Mandibular molar fragment; Likely Tribe Antilopini, Tribe Neotragini, or Tribe Oreotragini
3354	-	I	Mandibular molar fragment; Likely Tribe Antilopini or Tribe Oreotragini
3365	-	II/III	Horn core fragment with pedicle and sinus preserved
3417	Left	III	Mandibular i1
4002	-	II/III	Enamel fragment
4188	Left	II	Mandibular i1
4189	Left	II	Mandibular i3 or canine
4216	Right	III	Mandibular i3 or canine
4251	Right	-	Mandibular diastema fragment
4285	Right	I	Mandibular corpus fragment
4340	-	II/III	Horn core fragment from pedicle; Likely Tribe Alcelaphini
4374	-	-	Enamel fragment
4392	-	II/III	Horn core fragment
4440	-	-	Horn core fragment
4475	-	II/III	Horn core tip fragment; Likely Tribe Alcelaphini or Tribe Tragelaphini
4480	-	-	Possible horn core fragment

DN Specimen	Element Side	Size Class	Element Description
4495	-	II	Mandibular m1 or m2; Possibly Tribe Antilopini
4654	-	-	Enamel fragment
4655	Right	I	Mandibular diastema fragment preserving symphysis and alveolus for i3 and canine
4658	Right	5	Mandibular i1 or i2
4684	Right	3	Partial basioccipital
4704	-	-	Horn core fragment
4734	Left	II	Mandibular i1
4766	-	II/III	Maxillary molar fragment; Likely Tribe Alcelaphini
4771	Right	III	Maxillary molar fragment
4772	Right	II	Mandibular i1
4774	-	-	Enamel fragment
4797	-	II/III	Horn core fragment
4798	-	II	Partial basioccipital
4808	Right	II	Mandibular i3 or canine
4809	Right	I	Mandibular molar, possible m1; Likely Tribe Neotragini or Tribe Oreotragini
Postcranial			
1	Right	I	Humeral diaphysis with unfused metaphyseal regions
35		II	Partial proximal phalanx
38		II	Partial proximal phalanx
62	Left	III	Partial humerus with proximal epiphysis and diaphysis portion
65	Right	I	Radius distal epiphysis with unfused metaphysis
75	Right	I	Partial ulna proximal epiphysis lacking the olecranon
76	Right	I	Partial indeterminate metapodial distal epiphysis
79		II	Immature partial naviculocuboid
116		I	Partial ungal phalanx
116		I	Partial ungal phalanx
123		I	Partial indeterminate distal metapodial epiphysis
130		II	Partial intermediate phalanx
131	Right	II	Partial tibia with proximal epiphysis and diaphysis portion
132	Left	II	Immature partial calcaneus

DN Specimen	Element Side	Size Class	Element Description
136		II	Partial indeterminate metapodial distal epiphysis
147		II	Partial proximal phalanx
150		II	Partial indeterminate metapodial distal epiphysis
160		I/II	Partial metacarpal with unfused distal epiphysis
162	Right	II	Immature partial tibial proximal epiphysis with diaphysis
166	Right	II	Immature partial calcaneus
167		I/II	Partial proximal phalanx
182		I/II	Partial ungal phalanx
197		II	Partial proximal phalanx
200		I	Immature femur proximal epiphysis
207		II	Immature partial indeterminate metapodial distal epiphysis
228	Right	II	Partial metatarsal proximal epiphysis
239	Right	I/II	Partial ischium with acetabulum
277	Left	II	Partial astragalus
281		II	Partial intermediate phalanx
283		I	Partial ungal phalanx
304		I/II	Partial metacarpal distal epiphysis
320		I/II	Ungal phalanx
326		III	Partial intermediate phalanx
338	Left	II	Partial naviculocuboid
359	Right	I/II	Partial pubis with acetabulum
360		II	Immature partial intermediate phalanx
366		I/II	Partial ungal phalanx
377		II/III	Partial proximal phalanx
394		II	Partial ungal phalanx
444		II	Partial immature proximal phalanx
449	Left	I	Partial immature humerus distal epiphysis
462		II	Partial indeterminate metapodial distal epiphysis
467	Right	I/II	Partial metacarpal proximal epiphysis
473		II	Partial proximal phalanx
495	Right	I/II	Partial humerus proximal epiphysis

DN Specimen	Element Side	Size Class	Element Description
500		I/II	Partial ungal phalanx
510	Right	II	Partial metatarsal
511		II/III	Immature partial indeterminate metapodial distal epiphysis
514		II	Partial intermediate phalanx
537		I/II	Partial intermediate phalanx
538	Right	II	Immature partial calcaneus
553		II	Partial ungal phalanx
588	Left	I/II	Partial humerus distal epiphysis and diaphysis
589	Right	II	Partial astragalus
640	Right	III	Immature partial proximal calcaneus
642		I/II	Immature partial metacarpal distal epiphysis
711	Left	II	Partial metatarsal proximal epiphysis
715		II	Partial thoracic vertebra
716	Left	I	Immature partial femur proximal epiphysis
721		I	Partial intermediate phalanx
723		II	Partial ungal phalanx
729	Right	II	Partial naviculocuboid
760		I/II	Partial ungal phalanx
798		II	Partial lumbar vertebra
806	Right	I/II	Partial humerus proximal epiphysis
808		III	Partial intermediate phalanx
816	Left	II/III	Complete radius proximal epiphysis with partial diaphysis
846	Left	II/III	Partial ischial ramus
852		III	Partial radius distal epiphysis in two articulating fragments
858		II	Partial thoracic vertebra
875		II	Partial thoracic vertebra
876			Immature partial metacarpal distal epiphysis
877	Left	II	Partial astragalus
892	Right	II	Partial naviculocuboid
893		I/II	Partial proximal phalanx
894	Left	II	Partial ulnar carpal

DN Specimen	Element Side	Size Class	Element Description
896	Right	II	Partial external cuneiform
909	Right	I	Complete metatarsal proximal epiphysis with diaphysis
917		III	Immature proximal phalanx
944		II	Partial proximal phalanx
948		I/II	Partial ungal phalanx
957	Left	I	Partial ischium
985		III	Partial intermediate phalanx
1030		IV	Partial ungal phalanx
1078	Right	II	Partial tibia proximal epiphysis in two articulating fragments
1079		II	Partial indeterminate metapodial diaphysis
1080	Right	I/II	Partial femur distal epiphysis
1081		II	Partial lumbar vertebra
1095		II	Partial metacarpal distal epiphysis
1100			Partial metapodial diaphysis
1103			Partial lumbar vertebra
1106	Left	II	Immature partial humerus proximal epiphysis
1111	Right	III	Two elements: Partial radial carpal and partial scapula glenoid fossa
1199		II	Partial proximal phalanx
2000	Right	III	Partial humerus epiphyses and diaphysis in three articulating fragments
2002	Left	II	Partial femur distal epiphysis
2016		II	Partial proximal phalanx in two portions
2023		I	Partial proximal phalanx
2023		I	Partial proximal phalanx
2024		II	Partial proximal phalanx
2027	Right	II	Glenoid fossa of scapula
2031	Right	II	Immature metacarpal proximal epiphysis
2034	Left	II/III	Partial metatarsal proximal epiphysis
2044		3	Partial cervical vertebra
2058	Left	II	Glenoid fossil of scapula
2062		III	Partial thoracic vertebra
2064		III	Partial thoracic vertebra (associated with DN 2064)

DN Specimen	Element Side	Size Class	Element Description
2074	Right	III	Partial radius proximal epiphysis
2078	Right	II	Partial humerus distal epiphysis
2087	Left	II	Partial humerus distal epiphysis
2109	Left	II	Partial femur distal epiphysis
2115		I/II	Immature partial intermediate phalanx
2136		II/III	Partial ungal phalanx
2137	Right	I	Partial ulna proximal epiphysis
2152		II	Immature partial proximal phalanx
2153		II	Partial indeterminate metapodial distal epiphysis
2154		II	Partial indeterminate metapodial distal epiphysis
2172	Left	I/II	Partial naviculocuboid
2172	Right	I/II	Partial radius distal epiphysis
2172		I/II	Partial proximal phalanx
2172		I/II	Partial proximal phalanx
2172		I/II	Partial proximal phalanx
2172	Right	I/II	Partial metatarsal proximal epiphysis
2172	Left	I/II	Partial metatarsal proximal epiphysis
2174		II	Partial indeterminate metapodial distal epiphysis
2182	Right	I	Partial pisiform
2184	Right	II	Immature partial tibia distal epiphysis
2218	Right	I/II	Partial calcaneus
2239		II	Partial cervical vertebra
2241	Right	I	Partial scaphoid
2258		II	Partial ungal phalanx
2289		II	Partial proximal phalanx
2300	Left	I	Partial distal tibia epiphysis
2304		II	Partial proximal phalanx
2305	R	II	Partial ilium
2319		I/II	Partial intermediate phalanx
2326	L	II	Partial scaphoid
2331	L	I	Partial humerus diaphysis

DN Specimen	Element Side	Size Class	Element Description
2337		II	Partial cervical vertebra
2340		II	Immature partial tibia distal epiphysis
2347		II/III	Complete distal sesamoid
2371		II	Partial metatarsal diaphysis
2372		II	Partial metatarsal diaphysis
2384		I	Partial intermediate phalanx
2386		II	Partial proximal phalanx
2387		I	Immature partial ilium
2392	Left	III	Immature partial tibia distal epiphysis
2395		II	Immature metatarsal distal epiphysis
2398	Right	I	Partial femur proximal epiphysis
2432		II	Partial ungal phalanx
2435		I/II	Partial ungal phalanx
2436		II	Partial thoracic vertebra
2449		I/II	Immature partial metapodial diaphysis
2450		I/II	Partial lunate
2464		III	Partial intermediate phalanx
2471		II	Partial metapodial distal epiphysis
2476		I	Partial proximal phalanx
2484	Left	III	Partial distal scapula epiphysis
2486		II	Partial proximal phalanx
2486	Left	III	Partial 2nd/3rd carpal
2503c	Left	II	Partial ulna proximal epiphysis
2506		I/II	Partial ungal phalanx
2516	Right	I/II	Partial scaphoid
2530		I/II	Partial ungal phalanx
2531		II	Partial proximal phalanx
2534	Right	II	Partial ischium
2545	Right	II	Partial metacarpal proximal epiphysis
2552		I/II	Partial proximal phalanx
2553		II	Partial proximal phalanx

DN Specimen	Element Side	Size Class	Element Description
2577		I	Immature partial intermediate phalanx
2581		II	Partial thoracic vertebra
2583		II	Immature partial proximal phalanx
2588		II	Partial intermediate phalanx
2589		I/II	Partial intermediate phalanx
2592	Right	II	Partial astragalus
2593	Right	II	Partial astragalus
2672	Left	II	Partial 2nd/3rd carpal
2730		II	Partial indeterminate metapodial diaphysis
2742		II	Partial intermediate phalanx
2743		III	Complete proximal sesamoid
2764	Left	II	Partial metacarpal proximal epiphysis
2766	Right	II	Partial metatarsal proximal epiphysis
2769		II	Partial indeterminate metapodial diaphysis
2770	Left	II	Partial distal femur epiphysis in two articulating portions
2774		III	Partial intermediate phalanx
2784		II	Partial indeterminate phalanx
2790	Left	II	Partial ilium and acetabulum
2836		II	Partial sacrum in two articulating portions
2858		II	Immature metapodial distal epiphysis
2860	Left	II	Immature distal femur epiphysis and diaphysis in two articulating portions
2873		III	Complete distal sesamoid
2879		II	Partial metatarsal proximal epiphysis
2909		III	Partial intermediate phalanx
2930		II	Partial ungal phalanx
2979		II	Partial proximal phalanx
3032		II	Partial proximal phalanx
3048		I/II	Partial indeterminate metapodial distal epiphysis
3075		II	Partial proximal phalanx
3127	Right	I	Partial ischium
3136		II	Partial proximal phalanx

DN Specimen	Element Side	Size Class	Element Description
3150		II	Partial lumbar centrum
3154		III	Partial 2nd/3rd carpal
3155	Right	II	Partial distal radius epiphysis
3156			Partial intermediate phalanx
3159	Left	I/II	Pubis and partial acetabulum
3182			Partial indeterminate metapodial diaphysis
3186			Immature indeterminate metapodial distal epiphysis
3189		II	Partial cervical vertebra in three portions
3197			Partial ungal phalanx
3202	Left	II	Partial distal humerus epiphysis
3208		III	Immature partial proximal phalanx
3213		II	Partial cervical vertebra
3214	Right	II	Partial proximal humerus epiphysis
3222		II	Partial cervical vertebra
3225	Left	I	Partial 2nd/3rd carpal
3236		II	Partial thoracic vertebra
3250		II	Partial intermediate phalanx
3263		II	Partial indeterminate metapodial distal epiphysis
3268	Left	I	Partial scapula distal epiphysis
3279		II	Partial ungal phalanx
3319		I	Partial indeterminate metapodial distal epiphysis
3343	Left	II	Immature partial humerus diaphysis
3345		III	Complete proximal sesamoid
3347		II	Immature partial proximal phalanx
3352		II	Partial intermediate phalanx
3364		II	Partial ungal phalanx
3366		II	Partial proximal phalanx
3373	Right	II	Partial metatarsal proximal epiphysis
3375		II	Partial indeterminate metapodial proximal epiphysis
3382	Right	III	Partial radius proximal epiphysis and diaphysis
3401		III	Partial indeterminate metapodial diaphysis

DN Specimen	Element Side	Size Class	Element Description
3418	Left	II	Partial tibia distal epiphysis
3420	Left	II	Partial calcaneus
3436		I	Immature partial proximal phalanx
4139	Right	I/II	Partial humerus diaphysis
4142		I	Partial indeterminate metapodial diaphysis
4147	Left	II	Partial tibia diaphysis
4159			Partial thoracic vertebra
4166		II	Partial proximal phalanx
4176		II	Partial metacarpal diaphysis
4182	Left	II	Partial humerus distal epiphysis
4184		II	Partial proximal phalanx
4187	Right	II	Partial pubis
4196		III	Immature metacarpal distal epiphysis
4197		III	Partial tibia distal epiphysis
4199		II/III	Partial pubis with acetabulum
4204	Left	II/III	Partial ilium
4215		II	Partial metapodial distal epiphysis
4217			Partial proximal phalanx
4224	Right	III	Partial astragalus
4231		I/II	Partial cervical vertebra
4232	Right	II	Partial humerus distal epiphysis
4234a		I	Immature partial proximal phalanx
4234b		I	Immature partial proximal phalanx
4234c		I	Immature partial intermediate phalanx
4234d		I	Immature partial intermediate phalanx
4234e		I	Immature partial ungal phalanx
4234f		I	Immature partial ungal phalanx
4234g		I	Complete proximal sesamoid
4241	Right	I	Partial tibia distal epiphysis
4249		II	Immature partial metapodial distal epiphysis
4256		II	Partial intermediate phalanx

DN Specimen	Element Side	Size Class	Element Description
4277		II	Partial metapodial distal epiphysis
4278		I/II	Immature partial ischium
4291	Left	II/III	Partial tibia distal metaphysis
4293		II	Partial proximal phalanx
4294		II	Partial intermediate phalanx
4295		II	Partial intermediate phalanx
4296		I/II	Partial ungal phlanx
4305		II	Partial intermediate phalanx
4306	Left	II	Partial astragalus
4310		II	Partial ungal phalanx
4316		III	Partial metapodial distal epiphysis
4338	Right	II	Complete naviculocuboid
4338	Right	II	Partial naviculocuboid
4349	Right	I/II	Partial ilium and acetabulum
4355	Left	III	Partial pubis and acetabulum
4359	Left	I/II	Immature partial humerus distal epiphysis
4360	Left	I	Partial radius distal epiphysis
4365		II	Immature partial ungal phalanx
4366	Right	1	Immature partial radius diaphysis
4368		II	Immature partial proximal phalanx
4371			Partial indeterminate metapodial diaphysis
4382	Left	I	Partial ischium
4395	Left	II	Partial metatarsal proximal epiphysis
4396		I/II	Immature partial intermediate phalanx
4397	Right	II	Partial femur distal epiphysis
4402	Right	III/IV	Partial ulna proximal epiphysis
4403		II	Partial metacarpal distal epiphysis
4405		I	Immature partial intermediate phalanx
4406		II	Two elements: Immature proximal phalanx and immature intermediate phalanx
4407		I/II	Partial intermediate phalanx
4408		III	Partial indeterminate metapodial distal epiphysis

DN Specimen	Element Side	Size Class	Element Description
4410		II	Partial indeterminate metapodial diaphysis
4412		III	Immature proximal phalanx
4413		II	Partial indeterminate metapodial distal epiphysis
4414		II	Partial proximal phalanx
4415		III	Immature partial proximal phalanx
4416		I/II	Partial indeterminate metapodial distal epiphysis
4417		II	Immature partial proximal phalanx
4418		II	Immature partial metatarsal distal epiphysis
4419	Left	II	Immature partial metacarpal
4420		II	Partial intermediate phalanx
4421		I/II	Partial intermediate phalanx
4422		II	Partial intermediate phalanx
4423		I	Partial metapodial distal epiphysis
4424	Right	III	Partial astragalus
4439		I/II	Immature metatarsal diaphysis
4444	Left	I	Partial ulna diaphysis
4455	Left	II	Partial ulna proximal epiphysis
4456	Left	II	Partial calcaneus
4459		II	Partial proximal phalanx
4470	Right	II	Immature complete humerus diaphysis with metaphysis
4473	Left	I	Partial astragalus
4477		I/II	Partial humerus distal epiphysis
4508	Right	II	Partial humerus distal epiphysis
4512	Right	I	Immature partial radius diaphysis with distal metaphysis
4520		III	Partial indeterminate metapodial distal epiphysis
4521		II	Immature partial proximal phalanx
4524	Right	II	Partial calcaneus
4555		II	Partial proximal phalanx
4607	Left	II	Partial metatarsal proximal metaphysis
4645		I/II	Immature metatarsal distal epiphysis
4650		II	Partial intermediate phalanx

DN Specimen	Element Side	Size Class	Element Description
4652	Right	II	Partial humerus diaphysis and distal epiphysis
4657	Right	II	Partial humerus proximal epiphysis
4663	Right	II	Partial calcaneus
4664		II	Partial metatarsal distal epiphysis
4665		II/III	Partial ungal phalanx
4666		I/II	Partial ungal phalanx
4667	Right	II	Partial tibia distal epiphysis
4668	Right	II	Partial astragalus
4670		I/II	Partial ungal phalanx
4671	Right	II	Partial metacarpal proximal epiphysis
4673		II	Partial intermediate phalanx
4675		II	Immature tibia distal epiphysis
4676		II	Partial proximal phalanx
4677		II	Partial ungal phalanx
4679	Right	I	Partial tibia distal epiphysis
4682			Partial ungal phalanx
4693		I	Immature partial proximal phalanx
4705	Right	II	Partial astragalus
4708		II	Immature partial proximal phalanx
4710			Partial thoracic vertebra
4711		II	Immature tibia distal epiphysis
4757		II	Partial scapula glenoid fossa
4760	Right	III	Partial tibia distal epiphysis
4763	Left	III	Partial humerus distal epiphysis
4799		III	Immature partial intermediate phalanx
4800		I/II	Partial ungal phalanx
4801		II	Immature partial proximal phalanx
4802	Left	III	Partial scaphoid
4803	Right	II	Partial astragalus
4805		II	Partial cervical vertebra
4811	Left	I/II	Partial pubis and acetabulum
